# The pleiotropic roles of adipocyte secretome in remodeling breast cancer

**DOI:** 10.1186/s13046-022-02408-z

**Published:** 2022-06-14

**Authors:** Xiaomei Zhou, Jun Zhang, Wenchang Lv, Chongru Zhao, Yu Xia, Yiping Wu, Qi Zhang

**Affiliations:** 1 Department of Plastic and Cosmetic Surgery, Tongji Hospital, Tongji Medical College, Huazhong University of Science and Technology, 1095 Jiefang Avenue, Wuhan, 430030 Hubei China; 2 Department of Thyroid and Breast Surgery, Shenzhen Qianhai Shekou Free Trade Zone Hospital, Shenzhen, 518067 China; 3 Department of Gynecological Oncology, Tongji Hospital, Tongji Medical College, Huazhong University of Science and Technology, Wuhan, 430030 China

**Keywords:** Breast cancer, Adipocytes, Secretome, Cytokines, Adipokines, Immune regulation

## Abstract

**Background:**

Breast cancer is the leading female cancer type and the cause of cancer-related mortality worldwide. Adipocytes possess important functions of energy supply, metabolic regulation, and cytokine release, and are also the matrix cell that supports mammary gland tissue. In breast cancer tumor microenvironment (TME), adipocytes are the prominent stromal cells and are implicated in inflammation, metastatic formation, metabolic remodeling, and cancer susceptibility.

**Main body:**

It is well-established that adipocyte secretome is a reservoir engaged in the regulation of tumor cell behavior by secreting a large number of cytokines (IL-6, IL-8, and chemokines), adipokines (leptin, adiponectin, autotaxin, and resistin), lipid metabolites (free fatty acids and β-hydroxybutyrate), and other exosome-encapsulated substances. These released factors influence the evolution and clinical outcome of breast cancer through complex mechanisms. The progression of breast cancer tumors revolves around the tumor-adipose stromal network, which may contribute to breast cancer aggressiveness by increasing the pro-malignant potential of TME and tumor cells themselves. Most importantly, the secretome alterations of adipocytes are regarded as distinctly important targets for breast cancer diagnosis, treatment, and drug resistance.

**Conclusion:**

Therefore, this review will provide a comprehensive description of the specific adipocyte secretome characteristics and interactions within TME cell populations, which will enable us to better tailor strategies for tumor stratification management and treatment.

## Background

Breast cancer is the most common female cancer type and the leading cause of cancer-related mortality worldwide [1]. Breast cancer is a highly heterogeneous entity with multiple tumor subtypes, accompanied by frequent alterations in genetics and malignant cellular behaviors [2]. Adipocytes are the major component of breast tissue, approximately constituting 90% of the stromal tissue. According to the anatomical distribution, the fully differentiated breast primarily consists of two cellular compartments: the epithelial cell zone with glandular and lobular alveolar differentiated units containing branching ducts, and the connective tissue zone, commonly referred to as the breast fat pad, which is composed primarily of adipose tissue (AT) [3]. In general, normal mature adipocytes are separated from epithelial cells by the basement membrane, thus limiting the possibility of heterotypic crosstalk between the two cell types. Breast cancer usually begins in the epithelial cells surrounding the ducts and lobular tissue of the breast. Extracellular matrix (ECM) and epithelial cell degeneration may lead to the eventual destruction of the basement membrane that separates epithelial and mesenchymal tissues for increased tumor aggressiveness [4].

**Fig. 1:**
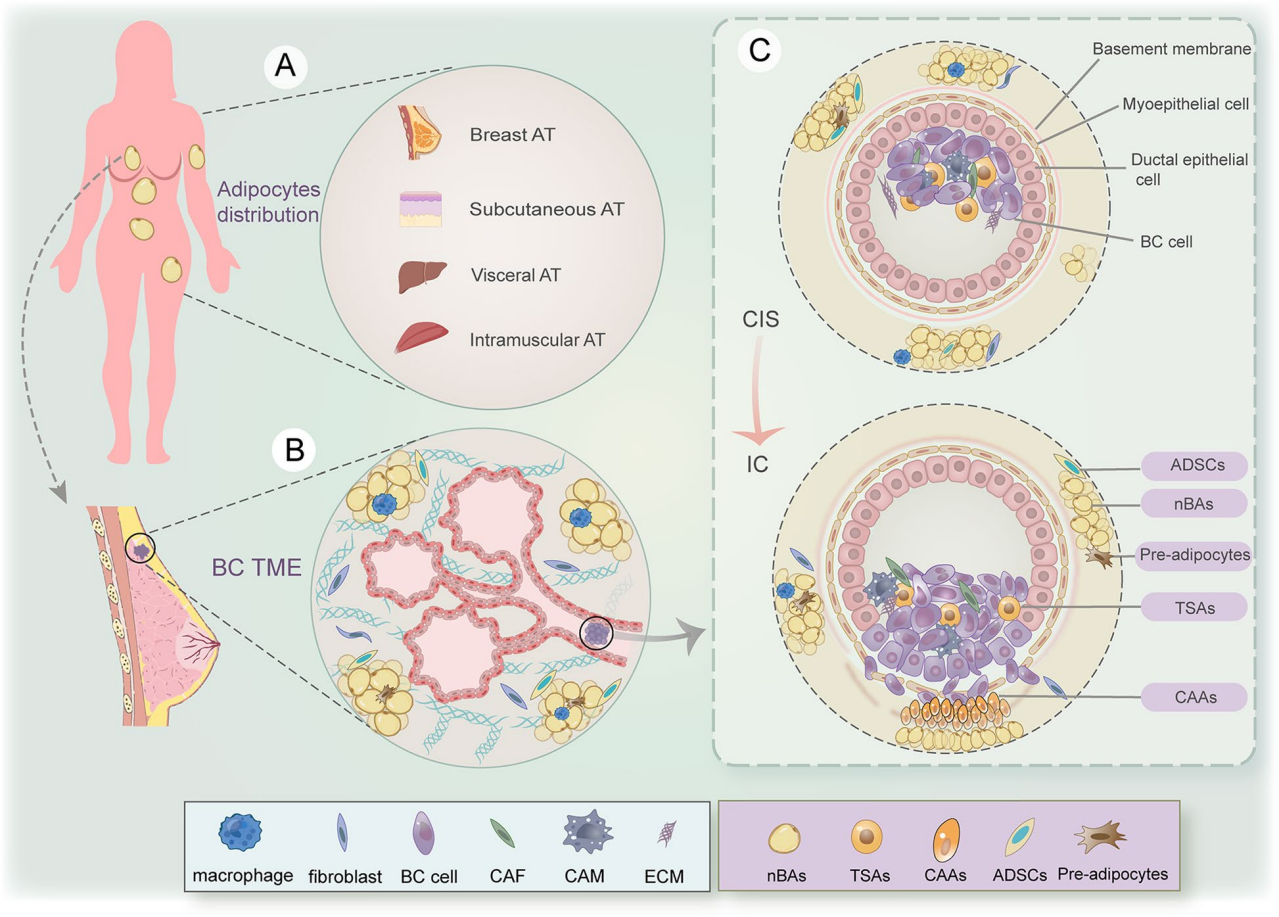
The adipocyte-containing tumor microenvironment (TME) in breast cancer (BC) tissue. **A** According to the distribution characteristics, adipose tissue (AT) can be cataloged into breast AT, subcutaneous AT, visceral AT, and intramuscular AT. **B** The adipocytes in breast AT (nBAs) represent a unique cell population to sustain the breast morphology. The main structure of the mammary gland consists of a system of acinus and lobules. **C** The TME in breast cancer consists of multiple adipocyte types, including ADSCs, pre-adipocytes, TSAs, and CAAs. Process of carcinoma in situ (CIS) and invasive carcinoma (IC) of the breast: the ducts consist of luminal cells (inner layer) and myoepithelial cells (outer layer), and are surrounded by the basement membrane, adipocytes, and other mesenchymal cells. CIS is characterized by tumor tissue distribution confined to epithelial cells, not breaking through the basement membrane, and not invading the interlobular space. Once the tumor cells have penetrated the basement membrane, invasive breast cancer occurs. At this point, breast cancer cells can interact with adipocytes at the invasion front, and educate them into CAAs to support breast cancer progression

The tumor microenvironment (TME) refers to the highly complex, heterogeneous, and spatiotemporally changing microenvironment in tumor entities, which profoundly and ubiquitously affects the biological behaviors and malignant evolutions of tumors [5]. The TME contains plentiful soluble secreted factors, coding and non-coding RNAs, and a wide variety of cell types, thus establishing an intricate niche for cellular crosstalk. Specifically, the types of cell components in the TME mainly include fibroblasts, immune cells, adipocytes, and vascular endothelial cells (ECs). Breast cancer cells can reprogram the TME through paracrine and autocrine, and altered TME in turn further promotes biological events, including breast cancer growth, invasion, angiogenesis, and metastasis [6–8]. Therefore, these series of related events lead TME to play a key role in supporting breast cancer development and tumor diagnosis and treatment. And very importantly, adipocytes are the most prominent cell type in the breast cancer TME (Fig. 1A-B). Adipocytes have been traditionally considered as energy storage cells, AT is broadly characterized by three storage types consisting of white, beige, and brown [9]. The endocrine, metabolic and inflammatory distribution of adipose depends on and is influenced by the estrogenic and androgenic status of AT [10]. The abundance of intratumoral adipocytes in breast cancer is implicated in inflammation, metastatic formation, metabolic changes, cancer stemness, and a supportive tumor immune microenvironment [11].

It is well-established that adipocytes can secrete tremendous factors engaged in the regulation of inflammation, metastatic formation, metabolic remodeling, and cancer susceptibility [12]. Specifically, adipocytes regulate tumor cell behavior either through cell-to-cell contact or through the release of proteins, lipids, metabolites, or exosomes, and vice versa. There are distribution regional, structural and functional differences among different types of adipocytes, including tumor-stromal adipocytes (TSAs), cancer-associated adipocytes (CAAs), normal breast adipocytes (nBAs), adipose-derived stem cells (ADSCs) and adipocytes in other parts of the body (Fig. 1C). TSA-breast cancer cell crosstalk has been recognized as a crucial event in facilitating breast cancer growth via paracrine release [13]. The breast cancer-neighbored aberrant adipocytes within invasive frontiers are denoted as CAAs. CAAs are characterized by smaller cell size, reduced lipid droplets, and a fibroblast-like phenotype, as well as reduced biomarkers of adipogenic differentiation, implying that these microscopic features of CAAs undergo dedifferentiation under the influence of neighboring cancer cells [14]. ADSCs possess multi-lineage differentiation potential and self-renewal capability, which is highly significant for developing regenerative strategies [15]. CAA-originated adipokines synergistically orchestrate the interaction between adipocytes, tumor cells, and other stromal cells [16]. Metabolically, adipocytes and ADSCs can fuel tumor growth by acting as energy reservoirs for adjacent breast cancer cells or by secreting key signaling molecules. A variety of physiological and pathological factors can trigger the changes in the morphology, quantity, and functional status of adipocytes. In the physiological status, pre- and post-menopausal, pre- and post-lactation, epinephrine level fluctuation, lymphedema status, and even hot and cold stimulation, are important factors affecting adipocytes [17–19]. Moreover, some specific disease states, such as mastitis, benign and malignant tumors, and endocrine diseases, can arouse persistent inflammation and hormone level disorders, which can cause AT inflammation, endoplasmic reticulum stress, ECM changes, and other mechanisms to cause adipocyte alteration and AT disorders [20]. In particular, epidemiological evidence highlights a strong association between obesity and higher cancer risk and poor prognosis in breast cancer patients [21]. Obesity makes the risks of breast cancer occurrence much more imminent. Adipose cachexia caused by obesity triggers malignant crosstalk between adiposity and tumors. The obesity-related adipocyte metabolism synthetically facilitates this condition by producing local and circulating pro-inflammatory secretome and initiating the malignant behavior of breast cancer cells [22] (Table 1).

**Table 1:** The morphology and functon comparison of nBAs, TSAs, and CAAs

	nBAs	TSAs	CAAs
**Definition**	Normal breast adipocytes far away from BC tissue	Adipocytes located at the stroma of tumor	Abnormal adipocytes located at the front of the tumor invasion
**Morphology**	Normal size, round shape, riched in lipid droplets that occupy 90% of the cell volume	Smaller size, spindle or ellipsoidal, reduced lipid contends	Dedifferentiated state, smaller size, fibroblast-like phenotype, smaller and reduced lipid contends
**Function**	Maintain the normal shape of the breast, normally secretion of diverse factors, possess energy storage functions and sustain energy balance	Important components of BC stroma, interact with BC cells and other stromal cells by releasing multiple adipokines, lipid metabolites and exosomes	Involve in malignant progression of BC by aberrantly secreted various cytokines, adipokines, lipid metabolites and exosomes
**Impact on BC**	The tumor-promoting effect is weaker than that of TSA and CAAs, and is the soil for BC cell seeds	Affect BC oncogenesis, proliferation and therapeutic outcomes in paracrine form by interacting within the complex network to support BC progression	Release molecules in promoting BC proliferation, migration, invasion, metastasis ability of BC cells by interacting with tumor cells, altered metabolic and immune status.

*Abbreviations*: *BC* Breast cancer, *CAAs* Cancer-associated adipocytes, *nBAs* Normal breast adipocytes, *TSAs* Tumor-stromal adipocytes

Therefore, the progression of breast cancer tumors revolves around the tumor-adipose network, which may promote breast cancer aggressiveness by increasing the pro-malignant potential of TME and tumor cells themselves. Here, this review emphasizes the pleiotropic roles of adipocyte secretome in remodeling breast cancer progression, including representative cytokines (IL-6, IL-8, and chemokines), adipokines (leptin, adiponectin, autotaxin, and resistin), lipid metabolites (free fatty acids and β-hydroxybutyrate), and other exosome-encapsulated substances, as well as their performance in vicious tumor-adipose interaction, immune regulation, and breast cancer diagnosis breast cancer therapy. The thorough understanding will provide novel perspectives on adipocyte-based therapeutic strategies for combating breast cancer.

## Adipocyte secretome in breast cancer progression

### Cytokines

#### Il-6

Interleukin-6 (IL-6) is a widely studied, multifunctional, and complex cytokine and is involved in pathophysiological processes such as immune response, inflammation, hematopoiesis, bone metabolism, and tissue repair [23]. Adipocytes are reverted to an immature and proliferative phenotype in the presence of breast cancer cells. Meanwhile, the cancerous CAAs more significantly promote the proliferation and migration in comparison to nBAs, by secreting the adipokines represented by IL-6 and monocyte chemoattractant protein-1 (MCP-1). Pre-adipocytes showed increased IL-6 secretion and expression of cancer-associated fibroblast (CAF) markers FSP1 and α-SMC and their pathological IL-6 overproduction led to the proliferation and migration of breast cancer cells and xenografts [24]. IL-6-mediated interactions between pro-adipocytes and breast cancer cells contribute to the progression of breast cancer.

Adipose stromal cells with plasticity could secrete IL-6 to promote migration and invasion of breast cancer cells, which could be mediated by cofilin-1 dependent pathway [25]. Lee et al. identified the pivotal role of mmu-miR-5112-Cpeb1-Il6 axis in promoting the inflammatory nature of CAAs in the breast cancer microenvironment [26]. Adipocyte-derived IL-6 and leptin enhanced the PLOD2 expression by activating the janus tyrosine kinase (JAK)/signal transducer and activator of transcription 3 (STAT3) and phosphatidylinositide 3-kinases (PI3K)/protein kinase B (AKT) signaling pathways, thereby promoting breast cancer metastasis in vivo [27]. Oncostatin M (OSM) is a pluripotential cytokine of IL-6 family members, and can inhibit interferon-β (IFN-β) expression and signaling in triple-negative breast cancer (TNBC) [28]. Lapeire et al. confirmed that cancer-associated AT boosted breast cancer progression by paracrine OSM and JAK/STAT3 axis activation [ 29]. Gyamfi et al. reported that paracrine IL-6 from matured adipocytes enhanced the aggressive epithelial-mesenchymal transition (EMT) behavior of breast cancer cells via inducing IL-6/STAT3 signaling [30]. Adipose-induced EMT in breast cancer cells can be reversed by inhibition of the IL-6/STAT3 signaling axis, by using U.S. food and drug administration (FDA)-approved anthelmintic niclosamide [31].

#### Il-8

Interleukin-8 (IL-8), also known as C-X-C motif ligand 8 (CXCL8), is a granulocytic chemokine with high expression levels in breast cancer [32]. IL-8 is responsible for immunosuppressive cell recruitment, inflammation, angiogenesis, and metastasis. Adipose stromal cell-derived IL-8 predominantly enhanced the breast cancer cell invasiveness and non-adherent growth ability in a paracrine manner [33]. CAA secreted a significantly higher IL-8 level and could enhance the EMT effect in normal breast luminal cells in an IL-8-dependent way [34]. Moreover, the ectopic expression of IL-8 in adipocytes in histologically noncancerous region activated them and enhanced their oncogenesis effects in a STAT3-dependent manner. This study also highlights multiple patterns of IL-8 inhibition that could effectively inhibit CAA features and their tumor-promoting effects. In addition, compared with single cultured breast cancer cells, the co-culture of adipocytes significantly enhanced the IL-8 secretion, which could increase the cell-adhesion molecules and influence the metastatic capacity of breast cancer cells [35]. Anti-IL-8 intervention altered the cytokine secretion diversion of breast adipocytes with reduced vascular endothelial growth factor (VEGF) secretion and affected the propagation of breast cancer.

#### Il-1β

Interleukin-1 (IL-1) is composed of two main isoforms, IL-1α and IL-1β, each of which is encoded by two separate genes [36]. IL-1β is a pro-inflammatory cytokine, and its abnormally high expression is a predictive biomarker of tumor malignancy and increased risk of bone metastasis from breast cancer [37]. IL-1β can be triggered by inflammatory signaling in multiple immune types and is an important factor and driver of tumor malignant evolution, therefore IL-1β blockade may be an important strategy to inhibit tumor growth and metastasis. Under obesity, IL-1β from primary adipocytes could induce the angiopoietin-like 4 (ANGPTL4) expression through activation of NF-κB and mitogen-activated protein (MAP) kinases, therefore promoting angiogenesis and tumor suppression [38]. Adipocyte-derived ANGPTL4 was beneficial to breast cancer angiogenesis and progression in obesity and was a potential therapeutic target for obese breast cancer patients.

#### CCL2

The MCP-1/C-C motif chemokine ligand 2 (CCL2) and its receptor interaction, including CC-chemokine receptor 2 (CCR2) and atypical chemokine receptor 2 (ACKR2) are renowned for their capability to drive the chemotaxis of myeloid cell and lymphocyte recruitment [39]. CCL2-CCR2 signaling axis function has various impacts on breast cancer progression, such as invasiveness and metastasis, recruitment of immunosuppressive cells, and stem cell self-renewal [40]. CAA-originated CCL2 participated in mediating the migration of ER+ and TNBC cells [41]. CCL2 produced by 3 T3-L1 cells was an important mediator in the crosstalk of adipocytes and 4 T1 cells [42, 43]. Anti-inflammatory drugs, such as aspirin and lunasin, can inhibit this crosstalk effect and thus prevent breast cancer progression. Liu et al. established a protein trap with CCL2 targeting ability to locally inhibit the CCL2 expression of tumor-associated adipocytes and thus lightened the immunosuppressive TME, which manifested as more abundant T cells and fewer M2 and myeloid-derived suppressor cells (MDSCs) [44]. Arendt et al. showed that in obesity, the enhanced production of CCL2 and IL-1β by the breast AT induced macrophage recruitment and crown-like structure (CLS) formation, which further induced angiogenesis and promoted the expanding tissue. This result demonstrated that the CCL2/IL-1b/CXCL12 was involved in the link of adipocytes and macrophages for carcinogenesis.

#### CCL5

CC-motif chemokine ligand 5 (CCL5) belongs to an important CC subfamily of chemotactic cytokines that can be secreted either by tumor cells or by stromal cells. CCL5/CCR5 combination is known for initiating and facilitating inflammatory responses, and acts as a tumor-promoting factor that is tightly associated with the invasive and metastatic stages of breast cancer [45]. The human adipocytes enhanced the invasiveness of MDA-MB-231 cells in the co-culture system [46]. This phenomenon was partially mediated by CCL5, and could be antagonized by using CCL5-blocking peptides and antibodies. Song et al. showed that the increased secretion of CCL5 in adipocytes enhanced the EMT effect of co-cultured TNBC cell lines, thereby promoting tumor growth and lung/liver metastasis in a mouse model [47]. Hence, selectively CCL5 targeted inhibition, such as emodin in adipose microenvironment might arouse positive therapeutic effects for breast cancer.

#### IGF-1 and IGFBP

Tumor stroma-derived insulin-like growth factor 1(IGF-1) and insulin-like growth factor binding protein (IGFBP) signaling have the potential to be novel targets for cancer therapy or adjuvant treatment, but these therapies have so far failed to yield significant efficacy [48]. IGFBPs show a tendency to be overexpressed and phosphorylated in several breast cancer subtypes, resulting in cancer cell proliferation, metastasis, and drug resistance-related properties [49]. The expression of IGF-1 content, which was regulated by glucose and fatty acids, was observably up-regulated in undifferentiated cells and differentiated adipocytes from obese individuals than those from lean individuals [50]. Correspondingly, the IGF-1 inhibition in adipocytes almost suppressed the growth-promoting effect of adipocytes on MCF-7 cells in vitro. The IGFBP-2 protein level in adipocytes was higher in adjacent peritumoral metastatic breast cancer tissues, compared to that in metastatic tissues [51]. Meanwhile, IGFBP-2 secreted by mature adipocytes greatly affected the metastatic capability of breast cancer cells.

#### VEGF

VEGF is an important angiogenic factor that initiates downstream signaling cascades by binding to VEGF receptors with different affinities and specificities, thereby stimulating tumor cell proliferation, growth, and neovascularization [52]. VEGF signaling also activates matrix metalloproteinase 2 (MMP2), matrix metalloproteinase 9 (MMP9), and urokinase plasminogen activator to degrade the ECM and basement membrane system [53]. Overall, VEGF affects the survival, proliferation, and migration of ECs, thus triggering angiogenic formation, liberating malignancy from the dormant phase, and inducing tumor vascularization.

Several studies have demonstrated the contribution of adipocyte-derived VEGF to the malignant progression of breast cancer. Lauriane et al. reported that the mature adipocytes, obtained after differentiation of human adipose-derived stem cells (hADSCs) from normal weight (MA20) and obese (MA30) women, were co-cultured with breast cancer cells to mimic the adipocyte microenvironment surrounding mammary tumors [54]. Compared with MA20, MA30 exhibited increased expression of leptin and cytochrome P450 family 19 subfamily a member 1(CYP19A1), especially VEGF. Supernatants from co-cultures of mature adipocytes and MCF-7 cells increased the proliferation, migration, and germination of human umbilical vein endothelial cells (HUVECs) independent of female body mass index (BMI). This promotive effect could be counteracted by neutralizing antibodies of VEGF and leptin. In addition, Francesca et al. found that the tumors co-injected with hADSCs grew faster and larger than those without hADSCs, accompanied by high vascularized biomarker VEGF and CD31 [55]. The evidence suggests that the pro-angiogenic effect of adipocytes in breast cancer may be partially mediated by VEGF.

#### G-CSF

Granulocyte-colony stimulating factor (G-CSF) is an important member of the hematopoietic growth factor family, mainly affecting neutrophil activation and regulation. G-CSF becomes aberrantly overexpressed in different types of cancer cells, such as lung cancer, gastric cancer, invasive bladder cancer, glioma, and a significant proportion of breast cancer [56]. G-CSF and neutrophils may play an essential role in synergistically promoting tumor metastasis. G-CSF is identified as one of the most highly up-regulated genes in CAAs and was increased in breast cancer-associated AT. Mechanically speaking, CAA-derived G-CSF was capable of promoting breast cancer migration and invasion via STAT3 signaling and other corporated CAA-derived factors IL-6 and granulocyte-macrophage-colony stimulating factor (GM-CSF) in vitro, thus forming an amplification effect to mediate breast cancer progression [57]. It posed a feasible strategy for interventing breast cancer invasivity by targeting the CAA-derived G-CSF axis.

### Adipokines

#### Leptin

Leptin is a key adipokine that is produced mainly by mature adipocytes. Leptin appears to be a key driver in energy homeostasis and breast cancer tumorigenesis, and its expression is associated with AT mass, BMI, and leptin receptor (OB-R) [58]. During the breast cancer progression, TSAs stimulated by external conditions can oversecrete leptin, which could bind to receptors on tumor cells and exert biological effects, triggering the effects on proliferation, migration, and tumor invasion [59].

Leptin was enriched in mammary adipocytes/AT, and notably suppressed CD8+ T cell function by activating the STAT3-fatty acid oxidation (FAO) axis and reducing glycolysis [60]. Targeting the leptin-STAT3-FAO axis might potentiate robust anti-tumor CD8 + T immune killing effect, in addition to inhibiting cancer stem cells (CSCs) and chemo-resistant tumor cells in breast cancer. Plasminogen activator inhibitor-1 (PAI-1) was a key effector of the metastasis in breast cancer cells co-cultured with adipocytes [61]. Then, adipocyte-secreted leptin promoted PAI-1-mediated breast cancer metastasis via activating the STAT3/miR-34a pathway in vivo. In addition, leptin promoted breast cancer cell migration and invasion via FAK-Src-dependent manner in vitro [62]. Kim et al. verified that leptin could enhance the cAMP response element binding protein (CREB)-dependent aromatase, which was related to the up-regulated cyclooxygenase-2 (COX-2) expression in breast cancer cells [63]. Wei et al. reported that caused the EMT effect of breast cancer via pyruvate kinase M2 (PKM2) up-regulation and PI3K/AKT signaling pathway activation [64]. Leptin derived from obese adipose stromal and ADSCs were highly expressed and promoted the proliferation and metastasis behaviors in the ER+ MCF-7 cells [65]. These studies suggest that functional crosstalk between leptin and estrogen signaling contributes to breast cancer development and progression.

#### Adiponectin

ADIPOQ/adiponectin is an abundant adipocytokine secreted by AT and adipocytes of breast cancer tissue and possesses insulin sensitizing, anti-inflammatory function, and antiatherogenic performances [66]. Epidemiological investigations have confirmed that breast cancer tumors in women with low circulating adiponectin levels exhibit more aggressive characteristics, such as higher histological grade and enhanced angiogenesis and metastasis [67]. Low circulating adiponectin level was a risk indicator for breast cancer patients regardless of postmenopausal status [68]. Compared with distant and node-negative sites, peritumoral adipokines were significantly altered, especially in the obesity setting and advanced breast cancer stage [69]. Macis et al. analyzed that in addition to enhancing the chemotherapy efficacy of breast cancer xenograft, the high expression of adiponectin was intensively related to the better overall survival (OS) in breast cancer patients with combined chemotherapy [70].

Many breast cancer cell lines, such as MCF-7, T47D, and SK-BR-3, could express adiponectin receptors AdipoR1 and AdipoR2 [71]. By binding to its receptor, adiponectin negatively the malignant behavior of tumors. Adiponectin could inhibit the leptin-promoted cell proliferation thus manifesting as a potent leptin antagonist [72]. Mechanically speaking, adiponectin inhibited the activation of leptin-mediated extracellular signal-regulated kinase (ERK) and AKT, and increased the leptin-inhibited protein tyrosine phosphatase 1B (PTP1B) expression and activation in vivo. Chung et al. deciphered that adiponectin promoted the autophagy-related cytotoxic death in breast cancer cells both in vitro and in vivo, which was mediated by STK11/LKB1-AMPK-ULK1 axis [70].

#### ATX

Autotaxin (ATX) is a secreted form of lysophospholipase D that yields extracellular lysophosphatidic acid (LPA) by hydrolyzing the choline of lysophosphatidylcholine (LPC) [73]. ADSCs and adipocytes, especially those adjacent to breast cancer tumor, were the major contributors to enhanced ATX level and plasma LPA in breast cancer patients [ 74, 75]. ATX-LPA signaling triggers key cellular events that lead to enhanced aggressiveness and motility of breast cancer cells.

The conditioned medium (CM) from mammary AT could enhance the ER-breast cancer cell proliferation in vitro, by promoting ATX secretion [76]. The bidirectional action between breast cancer cells and breast adipocytes modified the local ATX/LPA axis during the ER- breast cancer progression and increased ATX expression in the breast adipose pad. Cha et al. investigated that ATX-LPA signaling-related proteins, including LPA1, LPA2, and LPA3, were overexpressed in breast cancer adipose stroma, accompanied by abundantly infiltrating macrophages [77]. It indicated that ATX-LPA axis was a key coordinator in the inflammatory of adipose stroma. Benesch et al. verified that ONO-8430506 (an ATX inhibitor) efficiently reduced tumor growth by down-regulating inflammatory mediators in cancer-inflamed AT [75]. Therefore, the malignant inflammation cycle played an important role in breast cancer progression and could be interrupted by the inhibition of ATX.

#### Resistin

Resistin is acknowledged as a novel secretory factor from adipocytes, and may regulate inflammatory responses, affect tumor angiogenesis and modulate insulin sensitivity. Resistin is one of the highest regulatory adipokines secreted by 3 T3-L1 adipocytes under obesity-related metabolic settings compared to normal physiology. Lee et al. confirmed that breast cancer tissues, rather than adjacent normal breast tissues, possess an abundantly high expression of resistin, which level was positively correlated with tumor size, stage, lymph node (LN) metastasis, and ER status [78, 79]. High expression of resistin receptor CAP1 in tumors implied shorter OS, and relapse free survival (RFS) among ER+ tumors or LN-positive tumors [79]. Furthermore, resistin was proved to accelerate EMT and breast cancer cell stemness by toll-like receptor 4 (TLR4)-induced NF-κB activation [80].

Resistin could stimulate both ER+ breast cancer and TNBC cell proliferation and motility in vitro [79]. Lee et al. verified that resistin enhanced the phosphorylation of ezrin, radixin, and moesin via protein kinase C α (PKCα) [81]. This effect mediated the increased vimentin expression and facilitated breast cancer invasion. Gao et al. identified that resistin acted as the TAZ (a transcription cofactor) target to promote oncogenesis and was correlated with the adipocytic TAZ expression in TNBC tissue [82]. They suggested that the adipocyte-related TAZ/resistin signal was conducive to the development of breast cancer tumors, and the resistin neutralization represented a promising therapeutic strategy.

#### Visfatin

Visfatin/nicotinamide phosphoribosyltransferase (NAMPT) is an adipokine and an enzyme that is related to metabolic and immune disorders [83]. Human visfatin is predominantly produced by adipocytes and macrophages residing in AT. In addition, circulating visfatin concentrations are significantly higher in the obese state, and visfatin is negatively feedback regulated by insulin and leptin [84].

Hung et al. demonstrated that extracellular visfatin-promoted growth and lung metastasis of breast cancer were mediated by activating c-Abl and STAT3 [85]. Visfatin promoted the MMP2, MMP9, and VEGF gene expression and proliferation of MCF-7 cells [86]. Extracellular visfatin induced nicotinamide adenine dinucleotide (NAD), subsequently leading to the sirtuin 1 (SIRT1) activation and p53 deacetylation in vitro [87]. Wang et al. showed that pERK/CXCL1 activation was involved in visfatin-induced breast cancer progression in lung metastasis, which could be blocked by CXCL1 antibody [88]. In ADSCs and breast cancer cell co-culture, visfatin-preconditioned ADSCs (vADSCs) produced more activated tumor behaviors and tumorsphere formation [89]. In vivo, vADSCs promoted the tumor to form a larger entity. This result was mediated by visfatin-induced growth differentiation factor 15 (GDF15)-AKT activation in vADSCs.

#### MMP

Matrix metalloproteinases (MMPs) are regarded to be a category of proteins that mediate the degradation of the ECM of tumors and are primarily involved in tissue remodeling, inflammatory cell recruitment, and tumor invasion [90]. Thus, MMPs are important remodeling enablers of the TME. MMPs drive the malignant phenotype of breast cancer cells, including the acquisition of CSC characteristics and the induction of EMT. The overexpression of MMPs, especially MMP-9, MMP-2, and MMPs in serum, may indicate a higher risk of poor prognosis in breast cancer [91]. Leitner et al. demonstrated that osteopontin (OPN) and MMP were upregulated in obese AT [92]. OPN and MMP-cleaved OPN could both contribute to inducing aromatase activity and estradiol production in human adipocytes, and then possibly facilitated breast cancer cell proliferation.

#### Adipsin

Adipsin, known as complement factor D, can maintain AT homeostasis, and is primarily expressed in monocytes, macrophages AT, and ADSC within tumor tissue [93]. Goto et al. showed that adipsin originated from ADSCs distinctly promoted the proliferation and CSC-like features of human breast cancer patient-derived xenograft (PDX) cells [94]. Notably, hepatocyte growth factor (HGF) was secreted by mammary ADSCs and acted as a downstream molecular of adipsin in mammary ADSCs to improve the CSC properties in breast cancer [95].

#### Apelin

Apelin, also denoted as angiotensin-like-receptor 1 (APJ), is an endogenous modulatory peptide that is a ligand for the APJ receptor belonging to the G protein-coupled receptor family [96]. Besides, apelin is expressed in a large range of organs including brain, lung, kidney, pancreas, testis, prostate and AT [97]. Apelin expression is enhanced in various cancers, and the apelin/APJ axis has a crucial impact in tumor progress by reinforcing angiogenesis, metastasis, cell proliferation, and CSC plasticity and drug resistance. Gourgue et al. showed that apelin was an elevated adipokine in the subcutaneous adipose and tumor tissues of obese mice, while the apelin antagonist could inhibit the TNBC growth and brain metastasis formation [98]. In addition to obesity, the high tumor apelin expressions were related to a blunted response to N-acetylcysteine in their breast cancer cohort [99].

#### SFRP5

Secreted frizzled-related protein 5 (SFRP5) is considered to be a tumor suppressor gene and its circulating level is a feature in obesity-related metabolic disorders [100]. SFRP5 derived from 3 T3-L1 cells could suppress the migration and invasion of breast cancer cells by inhibiting Wnt and downstream EMT processes [101].

### Lipid metabolites

Adipocyte lipolysis could prepare metabolic substrates to fuel breast cancer cells, thus favoriting breast cancer progression [102].

#### FFA

Fatty acid (FA) is enriched in adipocytes, and could be transported to breast cancer cells. There is a complex metabolic symbiosis between adipose stromal cells and breast cancer cells that stimulate their malignant activity. After tumor-induced lipolysis, free fatty acid (FFA) released by adipocytes is transferred and stored in the tumor cells as triglycerides. FFA can be chronically released from lipid droplets via adipose triglyceride lipase (ATGL)-dependent lipolytic pathway [103]. In vivo, ATGL is expressed in human tumors, and its expression correlates with tumor aggressiveness and is upregulated by contact with adipocytes. Inhibiting the ATGL-dependent lipolytic/FAO pathway impeded the invasiveness of breast cancer cells induced by co-culture. Balaban et al. elucidated that breast cancer cells in an FA-rich environment were able to accumulate extracellular FA in the form of intracellular triacylglycerols (TAG), and the increased intracellular TAG levels, which could protect breast cancer cells from palmitate-induced apoptosis [104]. Zaoui et al. reported that the breast adipocyte secretome could promote malignant proliferation of breast cancer cells by enhancing CD36-mediated FA uptake. This function of breast adipocyte donors was unrelated to the state of BMI, menopausal status, and mammary density [105].

Byon et al. proved that FFAs could enhance PAI-1 expression in MDA-MB-231 cells and facilitate breast cancer progression in vitro, by partially inducing SMAD4 [106]. breast cancer cells affect lipolysis in adipocytes, and adipocytes affect β-oxidation in breast cancer cells [107]. Concretely, Adipocytes promoted breast cancer growth through mitochondrial β-oxidation by using fatty acid from adipocytes, of which process fatty-acid-binding protein 4 (FABP4) was a key activated target for metabolic interaction. Bellanger et al. adipocytes activated breast cancer cell autophagy by lysosome acidification, thus leading to tumorous survival and migration [108]. Adipocytes increased autophagic flux, autophagosome maturation, lysosomal acidification, and intracellular degradation in breast cancer cells, and that disruption of membrane phospholipid composition with reduced arachidonic acid content was responsible for adipocyte-induced activation of autophagy in breast cancer cells.

#### β-HB

Ketone body β-hydroxybutyrate (β-HB), is extracted from FAO and acts as a nutrient supply component for peripheral tissues [109]. Huang et al. identified that primary mammary gland-derived adipocytes could induce histone H3K9 acetylation and tumor-promoting gene up-regulation by secreting β-HB, thus increasing the malignancy of breast cancer cells [110].

#### ETP

Endotrophin (ETP) is a signaling molecule derived from collagen type VI and is richly expressed in AT [111]. Park et al. showed that ETP could promote collagen VI-stimulated tumor growth and TGF-β-mediated tissue fibrosis and EMT in tumor invasion [112]. ETP enhances fibrosis, angiogenesis, and inflammation by recruiting macrophages and ECs.

#### Lcn2

Lipocalin-2 (Lcn2), also denoted as neutrophil-gelatinase-associated lipocalin (NGAL), is a transporter protein related to energy homeostasis, inflammatory response, tumorigenesis, and tumor growth [113]. Drew et al. verified that reduced ERα expression in AT was associated with breast cancer proliferation and migration by increasing adipocyte-specific Lcn2 production and enhancing Lcn2 sensitivity of breast cancer cells [114].

## Adipocyte secretome in regulating immune cells in breast cancer

Complex interactions of environmental factors TME can have profound effects on the metabolic activities of immune cells, stromal cells, and tumor cell types. Macrophages are the most studied characteristic cells in adipose-tumor-immune cell interaction network. Crosstalk between adipocytes and macrophages profoundly affects breast cancer progression. In particular, the adipocyte-macrophage-breast cancer cell interaction in obesity involves multiple sequential mechanisms, including the release of inflammatory factors, ER stress, insulin resistance (IR), aromatase, and other hormone levels [17]. Focusing on tumor-associated macrophages (TAMs) and tumor-associated adipocytes and their crosstalk will provide new clues for breast cancer treatment.

Tumor-associated AT is typically featured by thin/fragile adipose membranes, necrosis, a high abundance of pro-inflammatory high-mobility group box 1 protein (HMGB1), as well as the loss of the lipid storage medium perilipin-1 [115]. The loss of adipocyte specification and necrosis directly activated macrophages and thus enhanced the inflammation state in TME. The combination of soluble breast adipocyte-derived leptin, insulin, IL-6, and TNF-α were inducers of the release of angiogenic mediators in macrophages, representing a potential mechanism for the enhanced risk of breast cancer progression in obese individuals [116]. Yadav et al. indicated that some adipocyte-derived mediators, such as leptin, insulin, IL-6, and TNF-α, could facilitate VEGF content in THP-1 macrophages in co-culture model. It confirmed the angiogenesis-promoting effect of adipocytes by enhancing the paracrine function of angiogenic factors of macrophage. Adipocyte-secreted IL-8 polarized the tumor-promoting neutrophils and increased the cell-adhesion molecules in breast cancer cells, thus facilitating the pro-inflammatory state in breast cancer [35]. Reciprocally, tumor-cell-derived factors and macrophage-related factors can also influence the inflammatory properties of adipocytes [43]. Targeting and reducing the inflammatory state associated with adipocytes is conducive to suppressing the progression of breast cancer with obesity.

Breast AT could intensify the breast cancer invasiveness via breast AT macrophages, and had a stronger relationship with breast cancer survival than breast tumor stroma macrophages [117]. There is an obesity-inflammation-aromatase axis in mouse models and female patients, which is featured by CLS [118, 119]. CLS is formed by macrophages surrounding dead or dying adipocytes and is denoted as a biomarker of the proinflammatory status of AT [120]. In the human cohort, CLSs of the breast are positively associated with older age, obesity, dyslipidemia, and higher levels of glucose, insulin, C-reactive protein, and IL-6 [121]. Macrophage-derived pro-inflammatory mediators induce aromatase and estrogen-dependent gene expression in adipocytes. Santander et al. verified that the AT of breast cancer tissue in obese mice presented a larger number of infiltrated macrophages, CLS, and hypertrophic adipocytes, compared with lean breast cancer tumor [122].

Leptin mediates the bidirectional action between breast cancer cells and TAMs. Leptin promotes migration and invasion of breast cancer cells by enhancing IL-8 expression in M2 macrophages [61]. Li et al. showed that leptin promoted the IL-18 expression and secretion in TAMs via NF-κB/NF-κB1 and breast cancer cells via PI3K-AKT/ATF-2 pathway, ultimately leading to breast cancer cell migration and invasion [123]. The adipocyte-released leptin could increase macrophage recruitment and activation by pleiotropic function regulation, such as various pro-inflammatory transcription factors and CD295 receptors in macrophages [122]. In Cha et al. study, CD68+/CD163+ macrophages were infiltrating and CLSs existed in AT surrounding breast cancer tissue. This specific macrophage and structures had a higher histologic grade of breast cancer [124].

In general, macrophages are a highly heterogeneous cell population with strong plasticity that communicates closely with adipocytes. Factors released by adipocytes, represented by leptin, insulin, and IL-6, can domesticate macrophages toward the function of promoting tumor malignancy, manifested by the changes in the secretion profile of macrophages, including pro-angiogenic VEGF and pro-inflammatory IL-6 [125]. On the other hand, the adipose-secreting profile can induce an increase in the number and activity of macrophages residing in ATT, which leads to the activation and amplification of inflammatory signals for favoring breast cancer progression. This functional alteration is closely relevant to the endocrine, paracrine, and autocrine pathways of adipocyte secretome [17]. Therefore, from the perspective of treatment, specifically targeting the function of macrophages in TME or reducing the number of immunosuppressive macrophages can block the progression of breast cancer by interrupting the communication between adipocytes and macrophages, promoting immune escape, and inhibiting angiogenesis. Of course, the role of the function and activity of other immune cells in the adipose-tumor interaction is also worthy of further investigation.

## Exosome-mediated horizontal transfer of adipocyte secretome in breast cancer

Exosomes are naturally occurring membranous extracellular vesicles (EVs) with 30–150 nm in diameter, characterized by conserved tetraspanins and key proteins associated with endocytosis and cargo sorting. Exosomes are a reservoir that contains genetic and functional molecules, such as nucleic acids (DNA, mRNA, ncRNAs), proteins, enzymes, and lipids. Functionally, exosomes are mediators of the interplay between adipocytes and tumor cells in breast cancer, and can promote the formation of the inflammatory microenvironment and systematically pre-conditioning metastasis niches for remote transmission and transfer [126]. Specific exosomal cargoes transported from AT might serve as endocrine or paracrine modes of intracellular communication between AT and other tissue types [127]. Exosomal mediators-dissociated from visceral adipocyte is capable of accommodating the key organ inflammatory and fibrosis signaling pathways [128].

EVs produced by metabolically abnormal adipocytes potentially fuel the progression of breast cancer. For instance, exosome proteins from IR adipocytes were associated with characteristics of EMT and CSCs in a cohort of breast cancer patients [129]. These adipocyte-exosomes carrying thrombospondin 5 (TSP5) dramatically enhanced the tumor EMT behaviors, and promote breast cancer malignancies in type 2 diabetes. hADSC-derived exosomes could induce MCF-7 migration by activating Wnt signaling in vitro [130]. Exosomes released by ADSC-differentiated adipocytes altered the transcriptome and improved MCF-7 growth via activation of Hippo signaling pathway [131]. And, the consumption of adipocyte exosomes weakened the tumor-promoting effect of adipocytes. Gernapudi et al. confirmed that exosomes secreted from preadipocytes modulated early-stage breast cancer via influencing stem cell renewal, breast cancer cell migration, and neoplasia through miR-140/SOX2/SOX9 axis [132]. Therefore, exosome-mediated horizontal transfer of adipocyte secretome induces breast cancer cells to exhibit metabolically reprogram and aggressive phenotypes both in vitro and in vivo [9]. Targeted exosome therapy is an essential approach to cut off the interaction between adipocytes and tumor cells and has potential for clinical therapy (Table 2).

**Table 2:** The role of adipocytes-derived exosomes in regulating BC progression

Exosomes sources	Exosomes contents	Target cells	Function and mechanisms	Ref.
IR adipocytes	TSP5	MDA-MB-231, MCF7, T47D	Induced EMT and CSC-related gene expression in BC cells, promoted BC malignancy in type 2 diabetes	[129]
hADSCs	N/A	MCF-7	Promoted MCF-7 migration by activating Wnt signaling in vitro	[130]
hADSCs	N/A	MCF-7	Promoted MCF-7 migration, reduced BC cell apoptosis and altered the transcriptome, promoted MCF-7 growth via activating Hippo signaling pathway	[131]
Pre-adipocytes	miR-140	DCIS cells	Influenced CSC renewal, BC cell migration, and neoplasia through miR-140/SOX2/SOX9 axis	[132]

*Abbreviations*: *BC* Breast cancer, *CSC* Cancer stem cell, *DCIS* Ductal carcinoma in situ, *EMT* Epithelial-mesenchymal transition, *hADSCs* Human adipose-derived stem cells, *IR* Insulin resistance, *TSP5* Thrombospondin 5

## Adipocyte secretome in breast cancer diagnosis

Some adipokines expression levels in serum, like resistin, leptin, adiponectin, and visfatin, can serve as risk factors for clinicopathological features of post-menopausal breast cancer, including tumor-node-metastasis (TNM) stage, tumor size, LN metastasis, and histological grade [133]. These adipokines are excellent candidates for surveillance and stratified management for postmenopausal breast cancer. Serum expression levels of leptin, resistin, and visfatin are reliable diagnostic markers of breast cancer and are independent predictive factors of LN invasion and ER-negative breast cancer patients [134].

Leptin and ObR are positively correlated, and both are highly expressed in primary and metastatic breast cancer tissue [135]. These two biomarkers are confirmed to be associated with poor prognosis, especially in G3 tumors. The high ratio of serum leptin/adiponectin might indicate the presence of aggressive breast cancer [136]. Lee et al. emphasized that resistin was a potential independent prognostic factor for breast cancer patients and for hormone therapy stratification and tumor therapy [78]. The epigenetic inactivation of secreted SFRP5 gene, especially SFRP5 promoter hypermethylation was associated with poor prognosis in breast cancer [137]. Afterward, the high level of SFRP5 in plasma and tumor tissue was related to better clinical factors in breast cancer patients, such as BMI and LN metastasis, TNM stage, as well as low high Ki67 expression [101]. Similarly, high serum endolipin and visfatin levels were linked to advanced tumor stage, greater tumor size, and poor outcomes in breast cancer patients, thus serving as a prognostic biomarker in breast cancer survival [85].

Histologically related adipokine staining is also a potential diagnostic method. There is a close connection between adipocyte hypertrophy and the staging and grading of breast cancer [138]. The staining assay showed that CCL5 expression located in peri-tumor AT was related to LN and distant metastases, and OS in TNBC patients [46]. Yamaguchi et al. underlined that AT invasion (ATI) at the tumor margin was very concerned about the lymph node involvement in patients with invasive breast ductal carcinoma [139]. Besides, ATI was related to patient age and invasive tumor size and acted as an excellent prognosis for this breast cancer subtype. Calligaris et al. found it was available to recognize the margin of breast cancer by utilizing desorption electrospray ionization mass spectrometry imaging (DESI-MSI) based on lipid profiles, since several fatty acids, including oleic acid, expressed higher levels in breast cancer tissue than in normal region [140]. This study supposed that the method based on lipid profiles was feasible for rapid intraoperative detection of residual breast cancer tissue during breast-conserving surgery.

## Adipocyte secretome in breast cancer therapy

Targeting these tumor-associated adipocytes may intervene or reverse the progression of breast tumors. Inhibition of these adipocytes can significantly impair breast cancer metastasis [43]. For instance, Alvarez-García et al. confirmed that melatonin could induce the decrease of the TNF-a, IL-6, and IL-11 gene expression in MCF-7 and 3 T3-L1 in their co-culture system [141]. Indeed, the appropriate intervention of specific adipocyte secretory factors described above may result in inhibition of tumor growth or metastasis.

The signatures of adipokines expression can affect the effect of hormone therapy in breast cancer patients. Tamoxifen efficacy could be antagonized by mature adipocytes derived from ADSCs of patients with obesity but normal-weight in vitro [142]. This was related to the elevated level of IL-6, leptin, and TNFα in adipose TME that counteracted on anti-proliferative efficacy of tamoxifen. The up-regulated IL-6 and FGF-2 abrogated or inhibited the anti-VEGF therapy in obese breast cancer patients and mouse model [143]. This effect could be reversed by IL-6 blockade through normalizing tumor vasculature and immunosuppression. Leptin sustained the resistance of aromatase inhibitor anastrozole in MCF-7 cells, thus leptin disturbance would be beneficial for patients with hormone-resistant breast cancer [144]. Adipocyte-secreted leptin also enhanced the HER2 protein levels through a STAT3-mediated up-regulation of Hsp90 in breast cancer cells in vitro [145]. Long-term exposure to leptin could markedly inhibit the sensitivity of breast cancer cells to the estrogen tamoxifen [146]. Meng et al. verified that in radiotherapy, AT developed an inflammatory wound-healing response and increased the secretion of ATX as well as other inflammatory cytokines, which could help breast cancer resistance to radiotherapy [147]. CSCs play an important role in tumor evolution and treatment resistance. Adipocyte-derived leptin was certified to propel the breast CSC self-renewal and paclitaxel chemoresistance via activating JAK/STAT3-regulated FAO in mouse breast cancer tumors in vivo [148]. Therefore, the secretion of adipocyte factors could endow drug resistance in breast cancer cells, by enhancing the release of inflammatory mediators and reducing FFA [149]. This process causes inflammation and lipid metabolic remodeling, and ultimately confers to the chemotherapeutic drug resistance.

## Future perspectives and challenges

Peritumoral tissue, stromal tissue, AT, and secreted adipokines have important roles in breast cancer biology. Adipocyte secretion profiles, especially TSAs and CAAs, are becoming critical contributors to breast carcinogenesis, diagnosis, and treatment. Currently, there are still many unresolved questions in terms of this field.

Firstly, there is a varied, dynamic, and complicated interplay between adipocytes and tumor cells. Breast cancer stimulation can induce adipocytes to revert to an immature and proliferative phenotype, thus in turn promoting breast cancer cell malignancy [41]. Adipocytes can also integrate the substances from the metabolic environment and promote the growth of breast cancer cells. Additionally, fatty acids released by adipocytes are taken up by breast cancer cells and they are utilized in the biosynthesis of new lipid compounds in breast cancer cells [150]. Adipocytes and breast cancer cells are competent to respond to endocrine and paracrine signals derived from both sides. Currently, most of the related studies have focused on the role of the adipocyte secretion profile in promoting breast cancer. However, some adipocyte factors have certain oncogenic effects or dual functions, thus playing multifaceted roles in different breast cancer processes and types. For example, oleic acid is deemed as an abundant monounsaturated fatty acid in the human body, which can release from adipocytes and affects different energy metabolism reactions [151]. The highly metastatic breast cancer cells could utilize oleic acid to sustain malignancy by AMPK activation-mediated β-oxidation. Nevertheless, oleic acid inhibited growth and survival in low metastatic MCF-7 cells in vitro [152]. Leptin influences the secretion of inflammatory cytokines in breast cancer cells, such as IL-1, IL-6, IL-17, and TNF-α, as well as the secretion of various growth factors and cytokines by the tumor stroma [153]. Obesity status leads to changes in the traits and functions of adipocytes, which in turn affects more aggressive changes in tumors. For example, Bowers et al. found that obesity-induced systemic IL-6 significantly increased the aromatase expression of pre-adipocytes through up-regulating breast cancer-derived prostaglandin E2 (PGE2) in postmenopausal patients [50]. In different metabolic environments, such as obesity, the precise mechanisms controlling these interactions are more complex. Targeting factors of adipocyte origin have the potential to be a useful approach to improving the prognosis of breast cancer patients in the setting of obesity.

Secondly, there is also a network of interactions between adipocyte factors that promote or constrain respective physiopathological functions. There were synergistic or antagonistic expressions among different factors. The expression of ADSC-derived IGF-1, HGF, VEGF, and IL-8 was higher in breast cancer tissue compared with the normal group [154]. Therefore, the diversiform ADSCs distributed in breast cancer entity release large amounts of tumor-promoting factors to support breast cancer development and evolution. The co-culture of breast cancer cells and primary human adipocytes enhanced VEGF and leptin secretion in adipocytes, and inhibition of VEGF in vitro also resulted in a dose-dependent decrease in leptin levels [155]. Therefore, it is a thorny and pressing problem to determine which secreted factor plays the main predominant function at a particular stage of tumor progression.

The development of novel sequencing technologies, including metabolomics, proteomics, single-cell omics, and spatial transcriptomics, can uncover potential adipocyte secretion profiles and key molecules that are expected to serve as potential diagnostic and therapeutic targets. Using scRNA-seq and snRNA-seq acting on adipose-specific knockout mice, will shed light on novel cell types or paracrine effects involved in the secreted signature of adipocytes [156]. The application of single-cell profiling in human AT can identify particular cell types for lipid storage and disease, including AT aging, adaptive thermogenesis, and tumor remodeling [157]. However, currently isolating intact and single adipocytes is complex due to their fragile characteristics. Similar sequencing strategies have not been thoroughly applied in human AT. But most certainly, these techniques will be instrumental in elucidating the broad range of molecular functions and adipocyte heterogeneity.

Given the complex role of the aforementioned adipocytes and factors in TME, targeting the pro-tumor pathways of these cells and factors, such as paracrine effects, metabolite reprogramming, and immunomodulatory effects, would be an effective way to combat breast cancer. However, targeting CAA or related components alone may not be able to completely ablate malignant cancer cells and seeds. More promisingly, a combination of cytostatic agents, specific factor inhibitors, and other conventional therapies will help to treat breast cancer.

## Conclusion

**Fig. 2:**
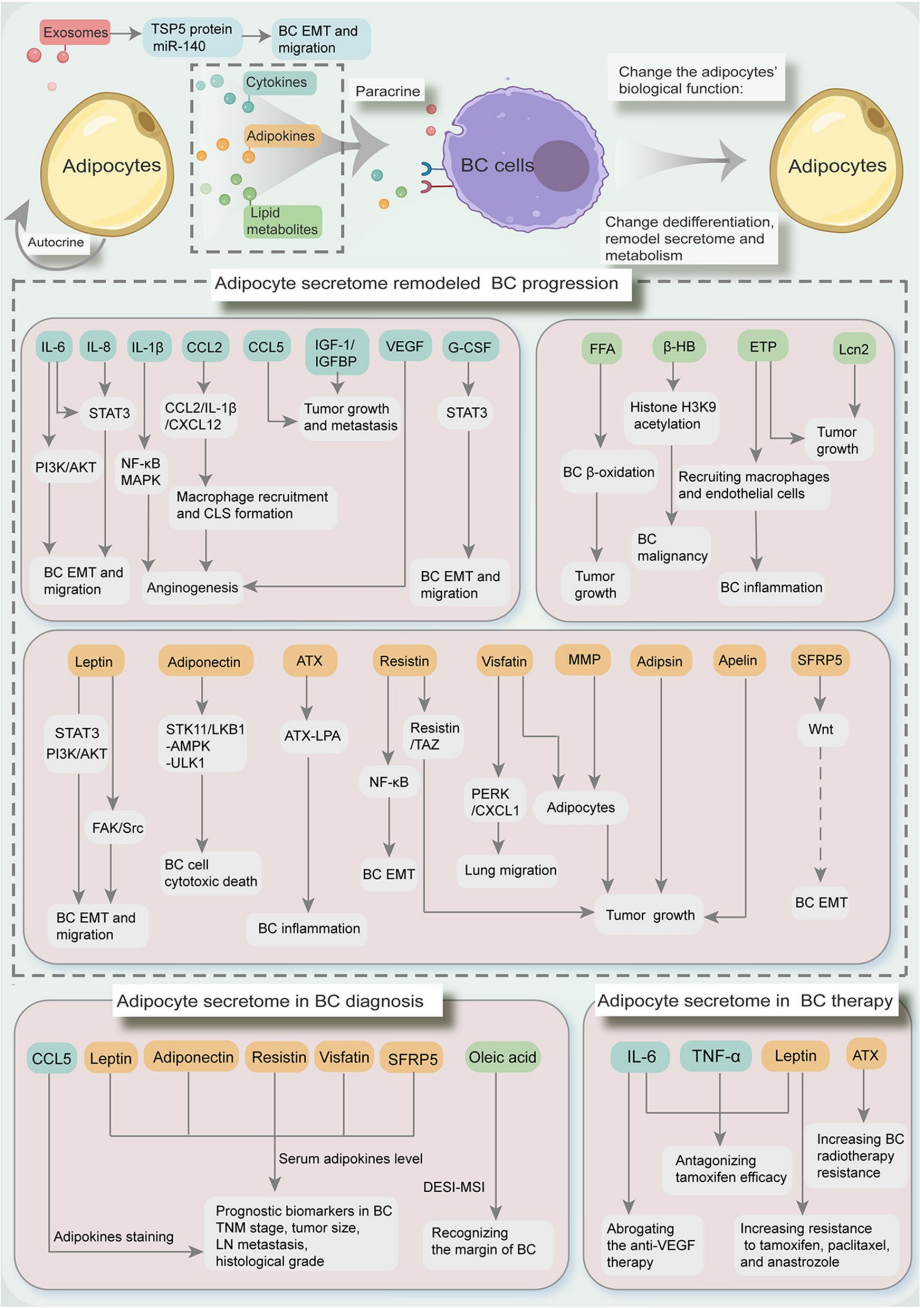
The pleiotropic roles of adipocyte secretome in remodeling breast cancer (BC). Adipocytes can affect themselves by autocrine and also affect or fuel tumors by paracrine. Reciprocally, breast cancer cells are also able to change the dedifferentiation, and remodel the function, secretome, and metabolism of adipocytes. The adipocyte-released secretome components, including cytokines (IL-6, IL-8, IL-1β, CCL2, CCL5, IGF-1, IGFBP, VEGF, and G-CSF), adipokines (leptin, adiponectin, ATX, resistin, visfatin, adipsin, MMP, apelin, and SFRP5), and lipid metabolites (FFA, β-HB, ETP, and Lcn2), profoundly impact on remodeling breast cancer progression and drug-resistance via multiple complicated mechanisms. Particularly, adipocyte exosomes are cellular vesicles to transport TSP5, miR-140, and other messages, leading to the altered behaviors of breast cancer cells

Adipocytes are phenotypically and functionally heterogeneous. In setting of breast cancer, adipocytes act as active facilitators, but not silent bystanders. There are pleiotropic roles of adipocyte secretome in remodeling breast cancer progression. The released cytokines, adipokines, and lipid metabolites from multiple adipocytes, are crucial orchestrators in shaping breast cancer growth, metastasis, immune regulation, exosome-mediated transfer, and breast cancer diagnosis and therapy (Fig. 2). The comprehensive understanding of specific adipose secretome characteristics and interactions within TME cell populations will enable us to better tailor strategies for tumor stratification management and treatment.

## Data Availability

Not applicable.

## Abbreviations

ACKR2: Atypical chemokine receptor 2
ADSCs: Adipose-derived stem cells
AKT: Protein kinase B
ANGPTL4: Angiopoietin-like 4
AT: Adipose tissue
ATGL: Adipose triglyceride lipase
ATI: Adipose tissue invasion
BC: Breast cancer
BMI: Body mass index
CAAs: Cancer-associated adipocytes
CCL2: C-C motif chemokine ligand 2
CCL5: CC-motif chemokine ligand 5
CCR2: CC-chemokine receptor 2
CLS: Crown-like structure
CM: Conditioned medium
COX-2: Cyclooxygenase-2
CREB: cAMP response element binding protein
CSCs: Cancer stem cells
CXCL8: C-X-C motif ligand 8
CYP19A1: Cytochrome P450 family 19 subfamily a member 1
DESI-MSI: Desorption electrospray ionization mass spectrometry imaging
ECM: Extracellular matrix
ECs: Endothelial cells
EMT: Epithelial-mesenchymal transition
ETP: Endotrophin
EVs: Extracellular vesicles
FA: Fatty acid
FABP4: Fatty-acid-binding protein 4
FAO: Fatty acid oxidation
FFA: Free fatty acid
G-CSF: Granulocyte-colony stimulating factor
GDF15: Growth differentiation factor 15
GM-CSF: Granulocyte-macrophage-colony stimulating factor
hADSCs: Human adipose-derived stem cells
HGF: Hepatocyte growth factor
HMGB1: High-mobility group box 1 protein
HUVECs: Human umbilical vein endothelial cells
IFN-β: Interferon-β
IGF-1: Insulin-like growth factor 1
IGFBP-2: Insulin-like growth factor-binding protein-2
IL-1: Interleukin-1
IL-6: Interleukin- 6
IR: Insulin resistance
JAK: Janus tyrosine kinase
Lcn2: Lipocalin-2
LN: Lymph node
LPA: Lysophosphatidic acid
LPC: Lysophosphatidylcholine
MA20: hADSCs from normal weight women
MA30: hADSCs from obese women
MAP: Mitogen-activated protein
MCP-1: Monocyte chemoattractant protein-1
MDSCs: Myeloid-derived suppressor cells
MMP2: Matrix metalloproteinase 2
MMP9: Matrix metalloproteinase 9
MMPs: Matrix metalloproteinases
NAD: Nicotinamide adenine dinucleotide
NAMPT: Nicotinamide phosphoribosyltransferase
nBAs: Normal breast adipocytes
NGAL: Neutrophil-gelatinase-associated lipocalin
OPN: Osteopontin
OS: Overall survival
OSM: Oncostatin M
PAI-1: Plasminogen activator inhibitor-1
PGE2: Prostaglandin E2
PI3K: Phosphatidylinositide 3-kinases
PKCα: Protein kinase C α
PKM2: Pyruvate kinase M2
PTP1B: Protein tyrosine phosphatase 1B
RFS: Relapse free survival
SFRP5: Secreted frizzled-related protein 5
SIRT1: Sirtuin 1
STAT3: Signal transducer and activator of transcription 3
STAT3: Transcription 3
TAG: Intracellular triacylglycerols
TAMs: Tumor-associated macrophages
TLR4: Toll-like receptor 4
TME: Tumor microenvironment
TNBC: Triple-negative breast cancer
TNM: Tumor-node-metastasis
TSAs: Tumor-stromal adipocytes
TSP5: Thrombospondin 5
vADSCs: Visfatin-preconditioned ADSCs
VEGF: Vascular endothelial growth factor
β-HB: β-hydroxybutyrate
